# Energy Metabolism and Brain Aging: Strategies to Delay Neuronal Degeneration

**DOI:** 10.1007/s10571-025-01555-z

**Published:** 2025-04-21

**Authors:** Donghui Na, Zechen Zhang, Meng Meng, Meiyu Li, Junyan Gao, Jiming Kong, Guohui Zhang, Ying Guo

**Affiliations:** 1 Department of Forensic Medicine, Hebei North University, Zhangjiakou, Hebei China; 2 Department of Pathology, Hebei North University, Zhangjiakou, Hebei China; 3 Department of Human Anatomy and Cell Science, University of Manitoba, Winnipeg, MB Canada; 4 Hebei Key Laboratory of Neuropharmacology, Hebei North University, Zhangjiakou, Hebei China; 5 Mudi Meng Honors College, China Pharmaceutical University, Nanjing, Jiangsu China

**Keywords:** Brain aging, Mitochondrial quality control, Neurons, Energy metabolism

## Abstract

Aging is characterized by a gradual decline in physiological functions, with brain aging being a major risk factor for numerous neurodegenerative diseases. Given the brain’s high energy demands, maintaining an adequate ATP supply is crucial for its proper function. However, with advancing age, mitochondria dysfunction and a deteriorating energy metabolism lead to reduced overall energy production and impaired mitochondrial quality control (MQC). As a result, promoting healthy aging has become a key focus in contemporary research. This review examines the relationship between energy metabolism and brain aging, highlighting the connection between MQC and energy metabolism, and proposes strategies to delay brain aging by targeting energy metabolism.

## Introduction

Aging is an inevitable process of a gradual decline in organ function and an increased susceptibility to diseases. The importance of healthy aging is highlighted by the World Health Organization's prediction that the global population aged 60 years and older will rise significantly between 2015 and 2025. At the cellular level, cellular senescence is marked by cell cycle arrest and is considered a major risk factor for age-related disorders, serving as a hallmark of aging (López-Otín et al. 2023). The bioactive substances secreted by senescent cells, collectively known as the senescence-associated secretory phenotype (SASP), contribute to chronic inflammation by affecting neighboring cells (Odawara et al. 2024). Neuroinflammation is one of the important factors contributing to neuronal damage and can also promote the inflammatory senescence of cells (Huang et al. 2022; Fulop et al. 2017). Correspondingly, senescent cells may also be a factor in persistent inflammatory reactions (Liu et al. 2024b). Current studies suggest that the onset of senescence can potentially be delayed (Yousefzadeh et al. 2018), although the specific mechanisms and strategies require further exploration. The question of how to delay aging or promote healthier aging remains a critical area of study. Crucially, advancing these efforts requires focused investigation into organs most vulnerable to aging, with the brain being a prime target due to its central role in maintaining both physiological and cognitive vitality.

The brain, an essential organ for regulating movement and cognitive processes, is particularly vulnerable to aging. Age-related diseases, which result in significant decline in memory and cognitive function, disproportionately affect the elderly (Chou et al. 2023). Unlike other organs, the energy metabolism of the brain involves complex cellular interactions. Although neurons make up only 2% of the body weight, they consume 70%–80% of the ATP in the brain and 20% of the glucose of the body (Rae et al. 2024). Neurons and astrocytes share an intimate connection in the realm of energy metabolism. The two collaborate metabolically to meet the brain's substantial energy demands. While neurons are traditionally considered post-mitotic cells, emerging experimental evidence has revealed aberrant cell cycle-related processes in specific subpopulations of excitatory neurons (Chow 2024). These findings suggest a potential mechanistic link between dysregulated neuronal cell cycle re-entry and age-associated neurodegeneration.

Understanding the relationship between the brain's energy metabolism and cerebral aging is instrumental in devising strategies to decelerate brain aging and mitigate related neurodegenerative diseases. This article primarily explores the energy metabolism between astrocytes and neurons within the brain, delving into the connection between cerebral energy metabolism and brain aging. Furthermore, we investigate mitochondrial dysfunction associated with neurodegenerative diseases and aging, summarizing several dietary strategies and their mechanisms of action. We provide a comprehensive assessment of their potential to delay brain aging.

## Nutrient Metabolism and Brain Aging

### Glucose Metabolism and Brain Aging

**Fig. 1:**
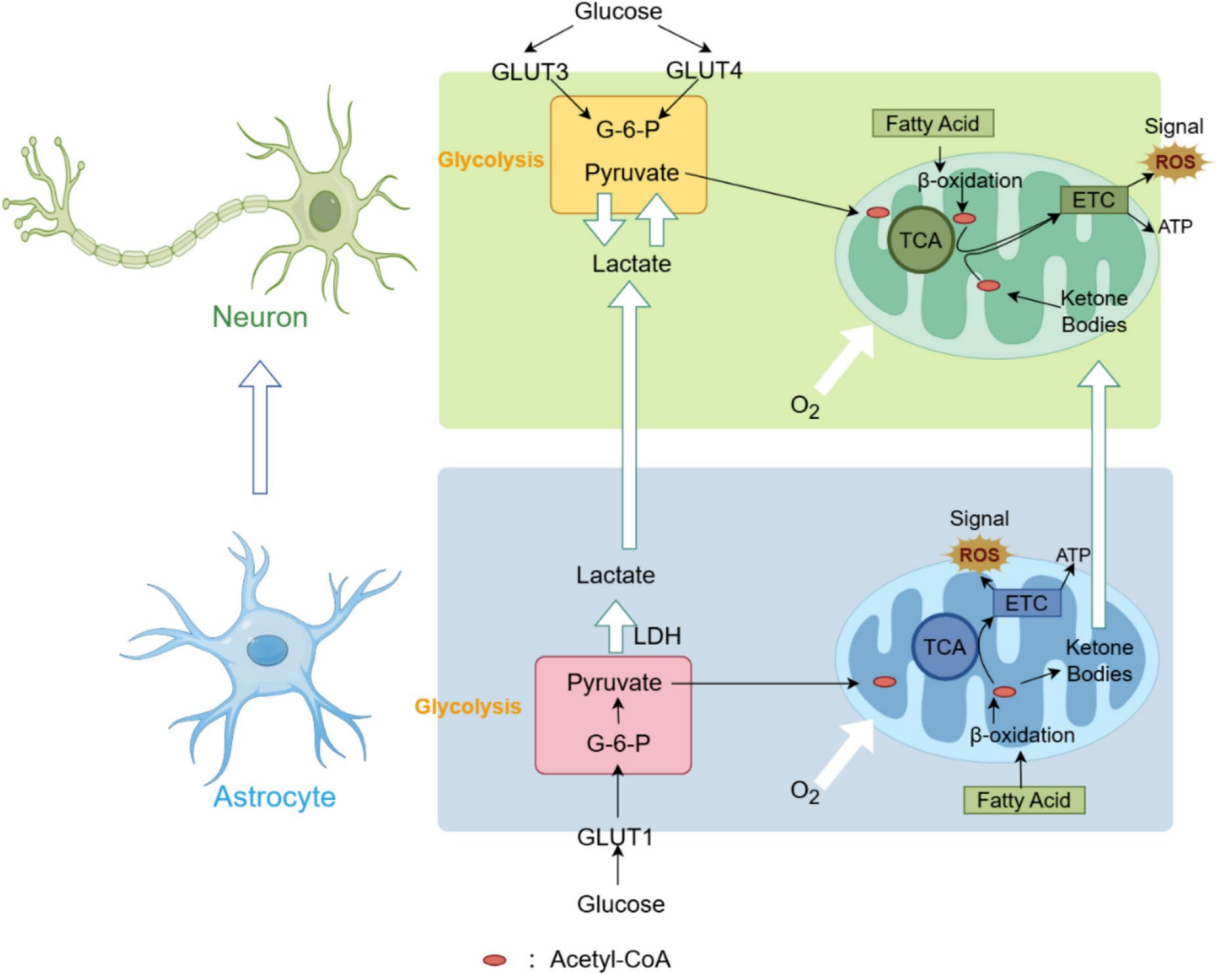
Neuronal energy metabolism is a complex process involving multiple interconnected pathways, primarily glycolysis, the tricarboxylic acid (TCA) cycle, and mitochondrial oxidative phosphorylation. Glucose uptake into neurons is mediated by specific glucose transporters, notably GLUT3 and GLUT4, located on the neuronal membrane. Following cellular entry, glucose undergoes glycolysis to yield pyruvate and a limited amount of ATP. Pyruvate is subsequently transported into mitochondria, where it is converted to acetyl-CoA via pyruvate dehydrogenase. This acetyl-CoA enters the TCA cycle, producing NADH and FADH2, which donate electrons to the electron transport chain (ETC) for ATP synthesis, while also generating reactive oxygen species (ROS) as metabolic byproducts. Cells can utilize ROS for physiological signal transduction processes. Beyond glucose metabolism, neurons can utilize alternative energy substrates. Dietary fatty acids are catabolized into acetyl-CoA through mitochondrial β-oxidation, with the resulting acetyl-CoA feeding into the TCA cycle to support oxidative phosphorylation. Furthermore, neurons benefit from metabolic coupling with astrocytes. Astrocyte-derived lactate, produced via glycolysis, is transported to neurons and converted to pyruvate for ATP generation. Similarly, ketone bodies generated from astrocytic fatty acid breakdown are delivered to neurons, where their conversion to acetyl-CoA fuels the TCA cycle, thereby maintaining neuronal energy homeostasis

Glucose metabolism is a crucial pathway for energy production in the brain, providing a significant portion of the ATP required for daily activities. Following glucose consumption, the membranes of neurons utilize glucose transporters to facilitate glucose uptake. Energy is generated through the tricarboxylic acid cycle, aerobic glycolysis in the cytoplasm, and oxidative phosphorylation in the mitochondria. Age-related impairments in glucose metabolism have been documented in studies (Yang et al. 2023). This section will explore the relationship between various glucose metabolism pathways and brain aging, identifying potential intervention strategies targeting specific aspects of these pathways (Fig. 1).

#### Glycolysis

In neurons, the main pathway for generating ATP is not glycolysis. However, due to its characteristic of generating ATP quickly, glycolysis can rapidly provide ATP for neurons. In the nervous system, astrocytes mainly use glycolysis as the pathway to produce ATP. The lactate produced by glycolysis in astrocytes can be transported into neurons and used to generate ATP. This process is called the astrocyte-neuron lactate shuttle (Zhang et al. 2023c). This theory has sparked discussions about neurons'main energy source. Recent studies have demonstrated that lactate can serve as an alternative energy source for retinal neurons, providing new insights into neuronal energy metabolism (Calbiague-Garcia et al. 2024). High lactic acid levels have also been found by research to be one of the markers of aging (Ross et al. 2010). Moreover, lactate is believed to function as a signaling molecule, regulating inflammation and calcium homeostasis in neurons and playing a crucial role in long-term memory (Alberini et al. 2018).

Ginsenoside-Rb1 (Gs-Rb1), a principal bioactive constituent isolated from Panax ginseng, has been extensively documented to exhibit multiple pharmacological activities, including anti-aging effects, anti-fatigue properties, and cognitive enhancement capabilities (Wen et al. 2023). It can increase sirtuin 3 activity, thereby benefiting glycolysis and local energy supply. In addition to analyzing the overall intensity of glycolysis in relation to aging, we can delve into specific steps of the glycolysis to explore their roles in aging process. Hexokinases (HKs), the key enzymes in the first step of glycolysis, exist in four isoforms. HK1, HK2, HK3 and HK4. Studies have found that overexpression of HK1 can delay the aging of mesenchymal stem cells (MSCs) by remodeling glycolysis (Sun et al. 2024b).While increased glycolytic flux ensures adequate ATP production, we propose that its overactivation might negatively impact the functionality of the pentose phosphate pathway. The underlying mechanism lies in the fact that these two metabolic pathways utilize glucose-6-phosphate (G6P) as their common substrate. Excessive glycolytic activity can lead to G6P depletion, thereby limiting its participation in the pentose phosphate pathway (Huang et al. 2024b; Tanvir et al. 2024), leading to reduced glutathione levels and a consequent impairment of the antioxidant capacity of neurons. Methylglyoxal (MG) is a highly reactive α-oxoaldehyde that is primarily generated intracellularly through the spontaneous degradation of glycolytic triose phosphate intermediates, namely dihydroxyacetone phosphate and glyceraldehyde 3-phosphate. MG can induce cellular dysfunction via chemical modification of proteins and lipids (Nokin et al. 2016). Studies have shown that reducing MG can slow down aging, indirectly highlighting the advert effects of excessive glycolysis on neurons (Uceda et al. 2024). In aging mice, knocking off the glycolysis-related essential enzyme 6-phosphofructo-2-kinase/fructose-2,6-bisphosphatase 3 gene reduces cognitive decline and synapse loss (Zhou et al. 2024). As the brain undergoes age-related degeneration, their ATP supply steadily declines. Enhancing glycolysis can help compensate for this deficit, potentially preventing neurodegenerative diseases and delaying neuronal aging. However, the potential drawbacks of increasing glycolysis must also be considered. Excessive glycolysis can reduce the levels of antioxidants in the nervous system (Herrero-Mendez et al. 2009; Flores-Ponce and Velasco 2024), and the accumulation of certain by-products of glycolysis can impair protein function. If glycolysis is excessively enhanced solely to meet ATP demands, it may ensure adequate ATP supply but could simultaneously weaken the antioxidant capacity of the nervous system. Further research is needed to develop strategies to stimulate glycolysis appropriately, ensuring that its byproducts are metabolized without accumulating in neurons, or to find ways to enhance glycolysis while clearing its harmful byproducts. This could be a promising direction for future research.

#### Tricarboxylic Acid (TCA) Cycle

TCA cycle, located in the mitochondrial matrix, is essential for metabolizing various substances. Acetyl-CoA then enters the TCA cycle, driving numerous metabolic processes. In neurons, acetyl-CoA serves as a direct substrate for ATP synthesis, and changes in its levels directly impact neuronal activity (Ronowska et al. 2018). Research has indicated that fluctuations in mitochondrial acetyl-CoA levels can influence cholinergic neuron transmission, affecting learning, memory, and cognition. Reduced acetyl-CoA levels decrease the carbon flow into the TCA cycle, subsequently lowering enzyme activity necessary for these reactions and reducing ATP synthesis (Paiva et al. 2024; Yao et al. 2023; Szrok-Jurga et al. 2023). In age-related neurological disorders such as AD, the catalytic activity of pyruvate dehydrogenase is suppressed, leading to decreased acetyl-CoA production and impaired TCA cycle energy metabolism (Sang et al. 2022). To address the decline in acetyl-CoA, strategies such as CMS121 and J147 have been developed. These compounds increase acetyl-CoA levels by inhibiting acetyl-CoA carboxylase I, thereby preserving mitochondrial homeostasis and alleviating symptoms of brain aging (Currais et al. 2019). However, a critical point to consider is that the analyses in this study were based on distinct brain regions. While their overall trends showed no significant discrepancies in interpretation, from a methodological perspective, it is advisable to select identical brain regions for investigation. This would enhance the comparability and interpretability of the effects of the two drugs. Within the TCA cycle, acetyl-CoA combined with oxaloacetate, catalyzed by citrate synthase, to form citrate. Previous mechanistic studies have demonstrated a significant association between citrate accumulation and lifespan extension with elevated citrate levels being consistently observed in aging model organisms. Recent research indicates that this accumulation of citrate exhibits sex-related differences, with increased citrate levels observed only in aging males (Navas-Enamorado et al. 2024). These changes in citrate levels suggest that the alteration of TCA cycle flux during aging may serve as a compensatory mechanism to address insufficient ATP production. Furthermore, the findings highlight the importance of considering gender differences when analyzing energy-related metabolites in aging organisms. However, excessive citrate can inhibit phosphofructokinase, a key glycolysis enzyme, thus preventing the synthesis of ATP (Tamarit-Rodriguez 2024). This suggests that when aiming to enhance ATP production by increasing TCA cycle flux, it is essential to consider the interplay between glycolysis and the TCA cycle, as excessive TCA cycle activity may suppress glycolysis. α-KG neutralizes hydrogen peroxide and other superoxide species, enhancing the activities of catalase, glutathione peroxidase, and SOD, thereby offering neuronal protection (Liu et al. 2018). In the hippocampal TCA cycle of aging β-amyloid precursor protein (APP)/Presenilin 1 (PS1) double transgenic mice, abnormal accumulations of citrate and succinate have been observed (Waddell et al. 2023). Reduced TCA cycle flow has been reported in postmortem brains of Alzheimer's patients, providing novel insights into early detection of brain aging or degeneration (Shao et al. 2023). This suggests two possibilities: aging animals exhibit abnormal TCA cycles, and these TCA cycle products could serve as novel aging markers.

Overall, while some TCA cycle products protect neurons, excessive citrate can inhibit glycolysis. Therefore, improving neuronal function through TCA cycle interventions requires careful consideration of the impact of TCA cycle to prevent disruptions in intracellular signal transmission.

#### Oxidative Phosphorylation (OXPHOS)

OXPHOS is the primary energy-producing process in the human body, occurring in the mitochondrial matrix. The electron transport chain (ETC), a crucial part of the OXPHOS, comprises four complexes: complex I (NADH dehydrogenase), complex II (succinate dehydrogenase), complex III (cytochrome c reductase), and complex IV (cytochrome c oxidase) (Tripathi and Ben-Shachar 2024).

While OXPHOS produces sufficient ATP to support cellular functions, it also generates ROS as byproducts. ROS, due to unpaired electrons, have strong oxidative potential and can damage cellular DNA and proteins (Guo et al. 2024; Jia and Sieburth 2021). Although small amounts of ROS are involved in physiological processes such as signaling pathways (Brieger et al. 2012). OXPHOS dysfunction can cause abnormal ROS buildup and mitochondrial malfunction, exacerbating various diseases, and the accumulation of ROS can accelerate aging (Finkel and Holbrook 2000). Reducing ROS levels has emerged as a potential therapeutic strategy to mitigate neuroinflammation and protect neurons. For instance, glutathione mitigates symptoms of AD by eliminating ROS and protecting neurons (Lana et al. 2024; Falcone et al. 2022). Extensive research has revealed that animal models with severe mitochondrial OXPHOS deficiencies consistently exhibit significantly shortened lifespans (Barends et al. 2016). At the cellular level, OXPHOS impairment triggers a cascade of pathophysiological events, most notably inducing a hypermetabolic state. This metabolic hyperactivity leads to concurrent mitochondrial DNA (mtDNA) instability and cell-autonomous stress responses, which are mechanistically linked to the development of a hypersecretory phenotype (Sturm et al. 2023). Crucially, there is a marked increase in telomere attrition rates with each cell division cycle under these conditions. Given that telomere length serves as a well-established biomarker of cellular aging (Zhu et al. 2019), these coordinated stress responses—encompassing metabolic dysregulation, genomic instability, and accelerated telomere shortening—may collectively accelerate aging process through multiple synergistic pathways., underscoring the critical role of OXPHOS in maintaining cellular health and preventing neurodegeneration.

Cellular senescence is characterized by a metabolic shift from OXPHOS to glycolysis, along with a disruption of this homeostatic equilibrium (Nolan et al. 2023). This pathophysiological transition suggests that pharmacological restoration of the OXPHOS-glycolysis axis could serve as a novel therapeutic strategy to ameliorate age-related bioenergetic decline through metabolic reprogramming interventions. Research has shown that ginsenoside Rg1 can prevent yeast cellular aging by balancing glycolysis and OXPHOS, regulating intermediates S. cerevisiae homologues of pyruvate kinase and succinate dehydrogenase iron–sulfur protein subunit (Wang et al. 2023). Hydrogen sulfide, as an ETC inhibitor, can also regulate the balance between OXPHOS and glycolysis (Na and Lee 2024). Studies indicate that in the presence of hydrogen sulfide, aerobic glycolysis increases while neuronal ATP production decreases (Wang et al. 2024). In addition to adjusting the intensity between OXPHOS and glycolysis to improve the energy supply of neurons, strategies for improving energy supply can also be found in the process of OXPHOS. Notably, interventions targeting the ETC—the core machinery of OXPHOS—have emerged as a pivotal approach to restore mitochondrial bioenergetics in neurodegeneration.

In fruit fly models of ALS, mitochondrial dysfunction could be attributed to defects in complexes I, III, and IV of the ETC (Nemtsova et al. 2023). The OXPHOS system undergoes age-dependent remodeling, characterized by marked reductions in protein abundance specifically in Complexes I, III, and IV. Notably, this molecular restructuring correlates with a pronounced age-associated decline in mitochondrial respiratory supercomplexes (mtRSCs) assembly, suggesting a potential mechanistic basis for progressive mitochondrial dysfunction during aging (Warnsmann et al. 2021). Alternate respirasomes, structurally comprising Complex I, dimeric Complex III (III2), and Complex IV, constitute autonomous functional units that integrate the complete electron transport chain while maintaining independent respiratory capacity (Gu et al. 2016). Enhanced assembly of these supramolecular structures demonstrates significant optimization of mitochondrial bioenergetics and augmentation of exercise performance (Champagne et al. 2016), with mechanistic studies revealing their critical role in minimizing electron leakage and suppressing ROS generation along respiratory chain components (Maranzana et al. 2013). However, a paradoxical finding emerges from recent investigations showing that severe depletion of alternate respirasomes fails to intrinsically impair basal oxidative phosphorylation efficiency (Milenkovic et al. 2023). This counterintuitive observation highlights fundamental uncertainties surrounding the homeostatic regulation of respirasome plasticity within cellular energy metabolism. Key persisting challenges include the absence of universally accepted definitions for functional respirasome configurations, incomplete understanding of stoichiometric prerequisites enabling efficient electron flux, and insufficient mechanistic insights into inter-complex crosslinking modalities that stabilize these macromolecular assemblies. Addressing these critical gaps demands an interdisciplinary approach integrating cryogenic electron microscopy (cryo-EM) for atomic-level structural resolution, spatially resolved crosslinking mass spectrometry to map dynamic protein interactions, and advanced metabolomic profiling to delineate real-time functional consequences of respirasome remodeling.

MPTP (1-methyl-4-phenyl-1,2,3,6-tetrahydropyridine) induces Parkinson's disease (PD) pathogenesis through selective inhibition of Complex I in the ETC (Gathings et al. 2024), while iron deficiency impairs neuronal development via suppression of mitochondrial complexes (Erber et al. 2022). These findings collectively demonstrate that discrete impairments in specific ETC components can propagate systemic dysfunction. This mechanistic understanding enables the development of targeted therapeutic strategies. For instance, PGC1α overexpression enhances Complex I and IV biogenesis, effectively ameliorating cognitive deficits in AD mouse models (Ng et al. 2025). Beyond direct modulation of respiratory complexes, emerging therapeutic paradigms should also address functional interactions within electron transport components, including mobile carriers and assembly factors that regulate ETC supercomplex formation. Coenzyme Q10 supplementation has demonstrated clinical efficacy in alleviating motor impairment associated with PD (Bagheri et al. 2023).

Thus, enhancing OXPHOS capability and increasing glucose utilization can protect neurons, decrease neuronal loss, and potentially delay brain aging. Future research may explore the combined use of antioxidants and medications to improve mitochondrial respiration, reducing ROS accumulation and increasing ATP production in aging neurons. However, extensive cellular and animal experiments are required to evaluate the safety and effectiveness of drug interactions, ultimately improving the quality of life for patients with neurodegenerative diseases and the elderly.

### Glucose Metabolism and Neurodegenerative Diseases

Building on the foundational overview of energy metabolism interventions in aging modulation, this section shifts focus to disease-specific metabolic dysregulation. Particular emphasis is placed on the pathological alterations in glucose metabolism observed across neurodegenerative disorders, including impaired insulin signaling and mitochondrial bioenergetic deficits. By systematically analyzing these metabolic perturbations, we subsequently delineate emerging therapeutic strategies that target distinct nodal points within cellular energy pathways—from glycolytic flux optimization to ketogenic metabolic reprogramming—which collectively aim to restore energy homeostasis and decelerate neurological deterioration.

In Alzheimer's disease (AD), a significant reduction in glucose metabolic capacity has been observed (O'Mahony et al. 2025; Pak et al. 2022), it’s an early sign that can manifest in the initial stages of AD (Grieb 2016). This phenomenon can be attributed to multiple pathological mechanisms, particularly the inhibition of cerebral glucose utilization mediated by Aβ-induced NOX2-dependent oxidative stress (Malkov et al. 2021). Notably, Aβ deposition represents one of the hallmark pathological features of AD. NOX2, a member of the NADPH oxidase family, catalyzes the conversion of oxygen molecules into superoxide anions, subsequently generating reactive oxygen species that exacerbate oxidative stress (Dustin et al. 2024). Furthermore, dysregulation of glucose transporters constitutes another critical mechanism. Postmortem studies in AD patients reveal significant reductions in cerebral GLUT1 and GLUT3 expression (Szablewski 2021), accompanied by decreased activity of key glycolytic enzymes such as hexokinase and lactate dehydrogenase (Wu et al. 2025). Research has shown that the GLUT inhibitor WZB117 can induce cytotoxicity, promote the production of Aβ peptides within cells, and increase the generation of ROS (Chandan et al. 2023). This finding highlights the potential severe consequences of GLUT dysfunction. Mechanistically, Aβ pathology has been shown to impair GLUT4 translocation, a process linked to cognitive decline and hippocampal hypometabolism (Pearson-Leary and McNay 2012). Notably, GLUT4—predominantly localized in the hippocampus and amygdala—is insulin-regulated and plays a pivotal role in neuronal energy homeostasis. Another critical point to emphasize is that type 2 diabetes mellitus (T2DM) is recognized as a significant risk factor for AD (Talbot et al. 2012). A hallmark feature of T2DM is insulin resistance, which refers to the diminished cellular response to insulin, leading to impaired glucose uptake and utilization (Zhang et al. 2025). Insulin plays a crucial role in signal transduction within the brain, particularly in processes related to learning and memory (Huang et al. 2024a). This suggests that insulin resistance may contribute to cognitive impairment, a phenomenon observed in the brains of AD patients. The underlying mechanism involves Aβ oligomers inducing neuronal insulin resistance through the activation of TNF-α and stress kinases such as JNK, IKK, and PKR (Mai et al. 2025). Furthermore, hyperglycemia resulting from insulin resistance can exacerbate cognitive dysfunction and activate microglia, leading to the sustained release of pro-inflammatory cytokines (Zeng et al. 2024). Additionally, insulin resistance may increase the risk of PD by altering metabolic processes and impairing the population of dopaminergic neurons (Zagare et al. 2025b). Emerging research indicates that insulin signaling dysregulation in GBA-associated PD (GBA-PD) is driven by the overexpression of FOXO1. Notably, knockdown of FOXO1 has been shown to increase the number of dopaminergic neurons in cerebral organoids derived from GBA-PD models (Zagare et al. 2025a). Targeting insulin resistance as a therapeutic strategy for neurodegenerative diseases may involve repurposing antidiabetic medications. For instance, metformin, a widely used diabetes treatment, has demonstrated neuroprotective effects on dopaminergic neurons in a rotenone-induced mouse model of PD (Wang et al. 2020). Additionally, insulin-sensitizing therapies such as pioglitazone, a thiazolidinedione-class antidiabetic agent, have shown promise in reducing Aβ deposition, attenuating neuroinflammation, and suppressing tau hyperphosphorylation (Yarchoan and Arnold 2014). These mechanisms contribute to the alleviation of diabetes-associated dementia (Ha et al. 2023; Bomfim et al. 2012).

While metabolic interventions targeting insulin signaling hold therapeutic potential, emerging evidence underscores the critical role of maintaining glycolytic flexibility—a parallel energy-generating pathway—to counteract neuronal bioenergetic collapse in aging-related neurodegeneration.

Impaired glycolysis can be detrimental to neurons, especially during high energy demand. In recent years, strategies for alleviating aging-related neurodegenerative diseases by targeting glycolysis have been widely studied. AZD5438 is a dual inhibitor of GSK3β and CDK. Studies have shown that AZD5438 can significantly enhance the glycolytic process (Shi et al. 2023), and this mechanism of action is likely achieved through the inhibition of the GSK-3 signaling pathway (Corona and Duchen 2015). This increase in glycolytic activity helps augment cellular energy supply, potentially exerting neuroprotective effects in the treatment of neurodegenerative diseases. Amyotrophic Lateral Sclerosis (ALS), a lethal neurodegenerative disease, affects both upper and lower motor neurons, leading to progressive muscle weakening and atrophy (Nguyen 2024). Superoxide dismutase 1 (SOD1), is a crucial antioxidant that shields cells from oxidative stress. Studies have demonstrated that impaired glucose absorption in the motor cortex of ALS patients and transgenic mice expressing WT or G93 A mutations of SOD1 exhibit lower mitochondrial respiration and ATP production (Hu et al. 2024). Improving glycolysis might mitigate early motor impairments in ALS by compensating for the reduced ATP production capacity (Manzo et al. 2019). AD is characterized by impaired glucose metabolism (Magi et al. 2021). Elevated lactate levels in the cerebrospinal fluid of AD patients suggest mitochondrial damage (Liguori et al. 2015). Enhancing brain energy metabolism can protect neurons and delay AD onset. However, excessive reliance on glycolysis can result in insufficient energy supply due to the high ATP demands of neural activity (Atlante and Valenti 2023).

In summary, the alterations in glucose metabolism observed in neurodegenerative diseases warrant significant attention, as targeting diverse glucose metabolic pathways may provide promising therapeutic strategies and potential intervention targets for developing comprehensive treatment regimens (Table 1).

**Table 1:** Changes in glucose metabolism in neurodegenerative diseases

Model types			Glucose metabolism alteration	Mechanisms
Mice		AD	Glycolytic activity is enhanced	Elevated H4 K12 la in Aβ-associated microglia boosts glycolysis by activating glycolytic gene promoters (Pan et al. 2022)
		AD	Low metabolism, increased glycolysis	Aβ activates HIF-1, driving glycolytic upregulation in AD pathogenesis (Yang et al. 2024a)
		APP^Swe^PS1^ΔE9^ model	Disruption of cerebral glucose metabolism	TREM1-activated microglia impair cerebral glucose metabolism (Wilson et al. 2024a)
		GLUT3cKO	GLUT3 KO neurons show reduced terminal glucose and ATP levels	GLUT3 deficiency impairs cellular glucose uptake, compromising glycolytic initiation (Li et al. 2023a)
		AD	Disruption of glucose metabolism and inhibition of glycolysis	Aβ and tau pathologies induce astrocytic IDO1 activation, subsequently translocating to the nucleus and suppressing glycolysis (Minhas et al. 2024)
		Aging‐associated sarcopenia	Decreased glucose uptake	PGC1α4 augments insulin signaling, facilitating GLUT4 translocation and enhancing cellular glucose uptake (Guo et al. 2023a)
		AD	Trem1 deficiency can restore brain glucose uptake	TREM1 suppresses the glucose-metabolizing PPP (Wilson et al. 2024b)
People	AD		Deteriorating brain glucose metabolism	The specific mechanisms have not been thoroughly investigated (Croteau et al. 2018)
	Aging		Inhibition of glucose metabolism	Inhibition of glycolysis, oxidative phosphorylation in adipose tissue (Xu et al. 2024)
	Aging		Deteriorating brain glucose metabolism	The specific mechanisms have not been thoroughly investigated (Nugent et al. 2014)
	ALS		Impaired glucose Tolerance	Increased FFA levels (Pradat et al. 2010)

H4 K12 la, histone H4 lysine 12 lactylation; HIF-1, hypoxia-inducible factor 1; APP^Swe^PS1^ΔE9^, amyloid precursor protein Swedish mutation with presenilin 1 delta E9 mutation; TREM1, triggering receptor expressed on myeloid cells 1; PGC1α4, peroxisome proliferator-activated receptor gamma coactivator 1-alpha isoform 4; PPP, pentose phosphate pathway; ALS, amyotrophic lateral sclerosis; AD, Alzheimer's disease; FFA, free fatty acid

### Protein Metabolism and Brain Aging

Proteins are one of the three essential nutrients vital for growth, repair, and regeneration. In eukaryotic cells, proteins are broken down into amino acids by proteases and lysosomes. Amino acids are classified as essential and non-essential. Previous research has shown that the leucine levels in the cerebrospinal fluid of the elderly are relatively high, potentially linked to their cognitive decline (Lin et al. 2021). In addition to leucine, other amino acids, such as alanine, valine, isoleucine, threonine, serine, proline, and phenylalanine, has been identified as potential markers for brain aging (Choi et al. 2024). Amino acids levels are closely related to the symptoms associated with aging, and certain amino acid precursors or their metabolites could potentially serve as indicators for assessing neurodegenerative diseases in the elderly (Gao et al. 2025). The following text explores the relationship between the metabolism of amino acids and brain aging.

Glu, the primary cationic neurotransmitter in the CNS (Li et al. 2024a), plays a crucial role in synaptic transmission. Upon its release into the synaptic cleft, Glu binds to specific receptors on the postsynaptic neuronal membrane, triggering specific intracellular changes (Puranik and Song 2024). Additionally, Glu can also be converted into the antioxidant glutathione and used as a carbon source for fatty acid synthesis (Zhang et al. 2024), this is helpful for improving the antioxidant capacity of the neurons. The levels of Glu in neurons are influenced by extracellular glutamate concentrations and its conversion in astrocytes. Recent studies have revealed that glutamine (Gln), a metabolic precursor of glutamate (Glu), plays a neuroprotective role in alleviating moderate cognitive impairment through maintaining the homeostasis of the Glu-Gln cycle (Baek et al. 2023). Although direct glutamate supplementation is clinically unfeasible due to its inherent neurotoxicity, Gln administration has emerged as a promising therapeutic strategy that supports neuronal energy metabolism while avoiding neurotoxic effects (Li et al. 2024c). Several approaches exist to supplement Gln, and studies have demonstrated that dietary supplementation with short-chain fatty acids (FAs) can delay the progression of AD in aging APP/PS1 mice by regulating Gln synthetase and enhancing the Glu-Gln cycle (Sun et al. 2023). Moreover, supplementing branched-chain amino acids in the diet of Tokai high avoider rats has been shown to improve learning and cognitive functions (Shida et al. 2021). These branched-chain amino acids, crucial suppliers of nitrogen for the Glu-Gln cycle, can directly impact the central nervous system's capacity for learning and memory, potentially reducing cognitive decline in aging (Suzuki et al. 2020). Additionally, Glu deamination can support cellular respiration, especially when glucose availability is limited, this indicates that Glu can also be one of the raw materials for energy metabolism (Zhang et al. 2024).

Notably, the interconnectedness of amino acid metabolic pathways extends beyond local neuronal energetics—systemic fluctuations in specific metabolites may reflect and even drive the progression of neurological aging. Beyond its neuroprotective role, the serum amino acid metabolic profile could serve as a biomarker of neurological aging (Rzepiński et al. 2022; Maszka et al. 2023). For instance, research by Chatterjee et al. discovered a significant correlation between metabolites in the kynurenine pathway (KP) and plasma Aβ levels in elderly individuals. Indoleamine 2,3-dioxygenase converts tryptophan to kynurenine via the KP pathway, and the concentration of Aβ in plasma can reflect its levels in the brain, contributing to the development of AD due to its aberrant deposition (Chatterjee et al. 2019). Neuroinflammation is also represented by elevated KP activity (Chen et al. 2010). Patients with mild cognitive impairment have been revealed to have abnormal neurotransmitter metabolism pathways. A portion of the cause can be traced to the decline in the brain's ability to produce neurotransmitters, which is intimately related to the reduced availability of corresponding amino acid precursors, such as methionine, tyrosine (required for the synthesis of dopamine and norepinephrine), and tryptophan (required for neurotransmitter) (Aquilani et al. 2023). Therefore, some amino acids or their metabolites not only function as neurotransmitters to protect neurons and enhance cognitive functions but also provide ATP to neurons during energy deficits. However, it is essential to carefully consider the types of amino acids supplemented to avoid potential neurotoxicity (Gruenbaum et al. 2024).

### Lipid Metabolism and Brain Aging

Lipid metabolism plays a critical role in aging. Dietary lipids are broken down by pancreatic or stomach lipases, producing free FAs. In neurons and astrocytes, there is not only a lactate shuttle but also a shuttle of ketone bodies (KBs). Notably, astrocytes are more efficient at oxidizing long-chain FAs than neurons. Astrocytes converts these FAs into KBs, which are then transported to neurons to serve as metabolism of energy (Panov et al. 2014; Li et al. 2024a). Morant-Ferrando et al. demonstrated that FA oxidation in astrocytes plays a crucial role in regulating energy adaptation and modulating cognitive function in murine models (Morant-Ferrando et al. 2023). While the energy yield from this metabolic pathway differs quantitatively from astrocytic glycolysis, it significantly contributes to maintaining astrocyte metabolic homeostasis and preserving ROS-mediated signaling transduction (Morant-Ferrando et al. 2023). The hypothesis regarding cerebral energy acquisition through FAs metabolism remains controversial, primarily due to the existence of rate-limiting enzymes in the FAs β-oxidation pathway within the nervous system (Antunes et al. 2024). Nevertheless, recent investigations by McMullen et al. have provided evidence that neuroglial cells can significantly augment neuronal energy metabolism through the coordinated utilization of FAs and KBs, especially under conditions of glycolytic impairment (McMullen et al. 2023). Therefore, some researchers have intervened in brain aging or neurodegenerative diseases related to aging by supplementing FAs or their metabolite KBs (Gu et al. 2024; Cao et al. 2024).

While these interventions demonstrate therapeutic potential, their efficacy is tightly regulated by metabolic context—a duality that underscores the need for precise spatiotemporal control of lipid utilization in aging neural circuits. Currently, supplementing with FAs or KBs has been shown to preserve neuronal function and promote neuronal metabolism. For instance, the ketone body β-Hydroxybutyrate (βOHB) has been observed to induce neuronal quiescence and enhance neuronal respiration, and potentially slow down the aging process by delaying the G1 to S phase transition (Koppel et al. 2023). Conversely, disruptions in OXPHOS can cause lipid accumulation in astrocytes, triggering neuroinflammation (Su et al. 2024). Emerging evidence indicates that the use of lipids as substrates for energy metabolism could potentially intensify oxidative stress, a critical aspect that warrants considerable attention in current research (Tracey et al. 2018). However, direct supplementation of polyunsaturated fatty acids (PUFAs) such as arachidonic acid, docosahexaenoic acid, and alpha-linolenic acid may extend lifespan and enhance resistance to hunger, oxidative stress, and heat stress (Qi et al. 2017). Omega-3 PUFAs exert beneficial effects on cellular energy metabolism by activating peroxisome proliferator-activated receptor alpha (PPARα), a nuclear receptor that enhances ATP production through the regulation of fatty acid oxidation (FAO) pathways. This PPARα-mediated metabolic modulation contributes to delayed cellular senescence and amelioration of age-related pathological alterations (Xiong et al. 2024). As a master transcriptional regulator, PPARα coordinates the expression of genes involved in FAO and autophagy pathways, thereby maintaining cellular metabolic homeostasis (Speeckaert et al. 2014). Furthermore, emerging evidence suggests that serine racemase, the enzyme responsible for converting L-serine to D-serine, plays a regulatory role in lipid metabolism within the subventricular zone of adult neurogenesis. This enzymatic activity potentially influences astrocyte-neuron metabolic coupling and D-serine biosynthesis, which are critically involved in the regulation of adult hippocampal neurogenesis (Roychaudhuri et al. 2023). This suggests that lipid metabolism may impact neuronal genesis, raising the possibility of using lipid metabolism-improving therapies to treat or slow the advancement of neurodegenerative disorders associated with neuronal genesis. When mitochondrial function is compromised, peroxisome FAs β-oxidation serves as an alternative ATP production pathway, particularly for metabolizing very long-chain FAs (Hammoud et al. 2024). In AD, Large amounts of Aβ are absorbed and stored by astrocytes, leading to mitochondrial dysfunction. This dysfunction prompts an increase in peroxisomes in astrocytes, which oxidize lipid droplets induced by oxidative stress to maintain ATP levels for neuronal energy metabolism (Zyśk et al. 2023). This supports other research suggesting that peroxisome proliferation protects against Aβ-induced neurodegenerative alterations by promoting neuroprotective effects (Santos et al. 2005).

FAs confer neuroprotective properties via their metabolic derivatives, with KBs emerging as critical mediators. These bioactive metabolites serve dual functions as bioenergetic substrates for neuronal ATP synthesis and as regulators of redox homeostasis and neurogenic pathways. Collectively, these mechanistic insights position the pharmacological modulation of lipid metabolism as a promising therapeutic strategy against neurodegenerative pathologies.

### The Unique Aspects of Brain Metabolism

While the fundamental energy production pathways (glycolysis, TCA cycle, and oxidative phosphorylation) are evolutionarily conserved across cell types, the brain has evolved specialized metabolic architectures to sustain its extraordinary bioenergetic demands. Unlike peripheral tissues where energy production and consumption occur within the same cell, neural circuits exhibit a remarkable metabolic compartmentalization—astrocytes and neurons engage in tightly coordinated substrate exchange to maintain neuronal activity.

As the primary functional units of the brain, neurons exhibit exceptionally high energy demands. Their intrinsic energy production is insufficient to meet these demands, prompting extensive research into their energy supply mechanisms. One prominent hypothesis posits that lactate serves as a major energy substrate for neurons (Kim et al. 2025). Lactate, produced through glycolysis in astrocytes, is transported to neurons and converted into pyruvate, which directly enters mitochondrial energy production pathways (Pellerin and Magistretti 1994). Some studies suggest that lactate is a more efficient energy substrate than glucose, with evidence indicating that neurons generate energy more effectively when utilizing lactate (Dienel 2019). Consequently, lactate is increasingly regarded as a superior energy metabolite compared to glucose (Itoh et al. 2003). When neuronal glucose uptake is impaired, the astrocyte-neuron lactate shuttle assumes a critical role in supplying ATP. Although neurons are post-mitotic cells, the concept of neuronal aging has not been widely acknowledged. However, recent studies suggest that the brain harbors a diverse array of neuronal subtypes, some of which exhibit heightened susceptibility to aging (Welch et al. 2022). Nonetheless, the prevailing view in the field is that brain aging predominantly reflects the senescence of glial cells, which support and surround neurons (Tamatta et al. 2025). Therefore, the capacity of senescent astrocytes to provide sufficient lactate for neuronal energy metabolism, as well as their ability to effectively uptake glucose and store it as glycogen for subsequent lactate production, is of critical importance for neuronal energy supply (Proia et al. 2016). Current research findings demonstrate that glycogen storage in aged astrocytes shows no significant difference compared to that in young astrocytes, and they maintain the capability to normally supply lactate to neuronal axons (Bastian et al. 2022). In addition to providing lactate to neurons, astrocytes are also capable of supplying ketone bodies, which are derived from fatty acid oxidation within the astrocytic cytoplasm (Panov et al. 2014). This metabolic pathway is distinct from intracellular glycolysis, as fatty acid oxidation occurs in the mitochondria and generates moderate levels of ROS that contribute to cellular signaling processes (Liu et al. 2024a). Experimental evidence demonstrates that genetic ablation of carnitine palmitoyltransferase-1 A (CPT1 A), a key enzyme in mitochondrial fatty acid oxidation, specifically in adult mouse astrocytes, results in significant cognitive impairment (Morant-Ferrando et al. 2023). The relative contribution of these two metabolic pathways remains a subject of ongoing debate. While some studies suggest that astrocytes primarily support neuronal energy demands through glycolytic metabolism, other investigations have demonstrated significant mitochondrial oxidative metabolism in the adult human brain (Lovatt et al. 2007). Notably, fatty acid oxidation has been shown to contribute up to 20% of total brain energy expenditure (Ebert et al. 2003). In senescent astrocytes, mitochondrial fragmentation becomes markedly evident (Araujo et al. 2024), potentially indicating differential alterations in the intensity of fatty acid oxidation and glycolysis within aging astrocytes.

## Energy Metabolism and Mitochondrial Quality Control

The above text has introduced several energy metabolism pathways. Among them, the mitochondrion is the main site for energy metabolism. MQC refers to a series of regulatory mechanisms adopted by cells to maintain the normal functions and quantity of mitochondria. Should the desire arise to further explore the relationship between energy metabolism and aging, MQC can be used as a bridge connecting the two. Among several pathways of MQC, cells rely on mitophagy, which removes damaged mitochondria, and mitochondrial-derived vesicles (MDVs), which transport mitochondrial-damaged components to lysosomes or peroxisomes for degradation. The two ways are closely correlated with energy metabolism. Balancing energy production with MQC is crucial for maintaining cellular health. The following two parts will mainly introduce the relationships of mitophagy and MDVs with energy metabolism, providing targets and inspiration for formulating intervention programs to delay aging.

### Mitophagy in Energy Metabolism

**Fig. 2:**
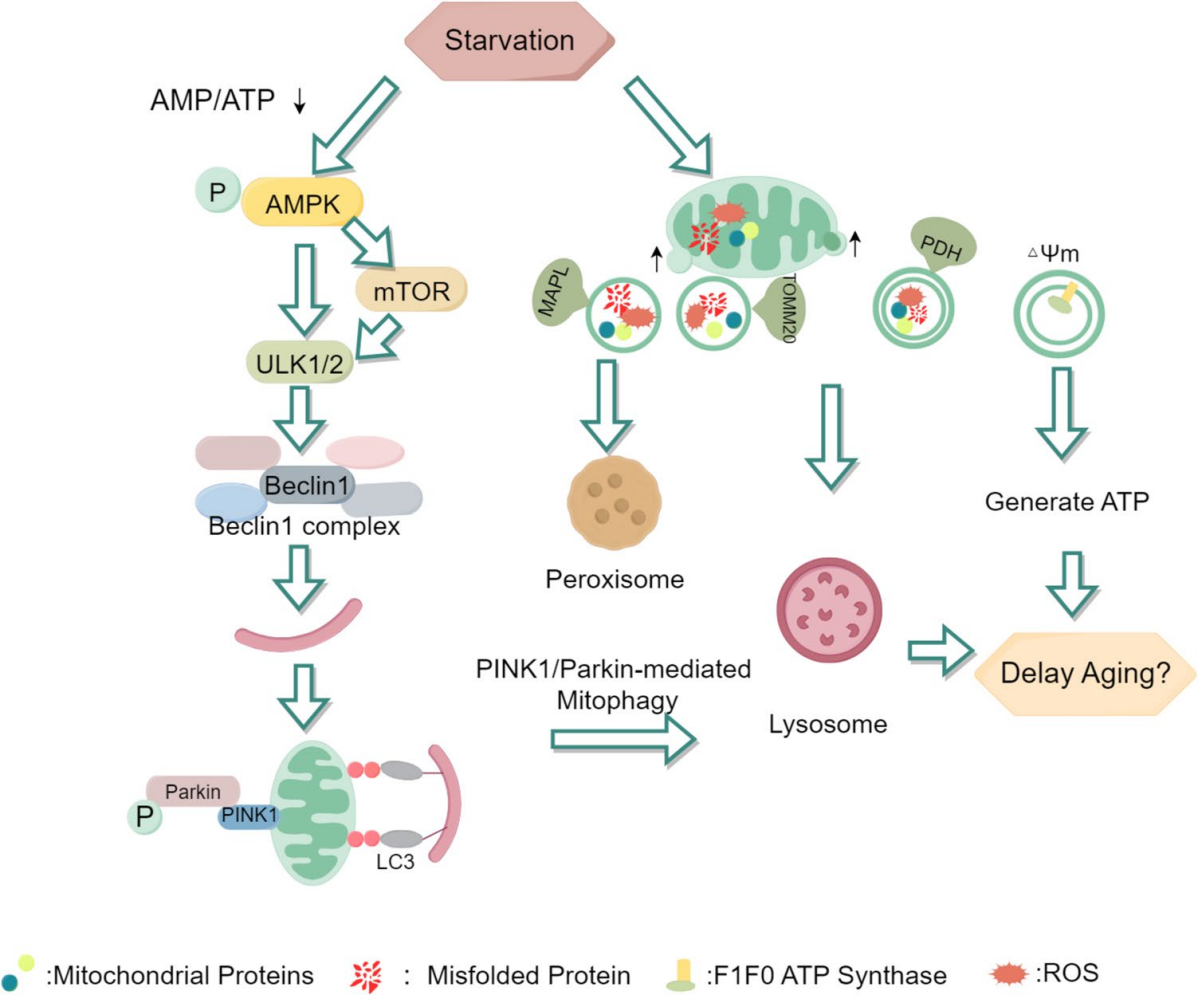
Starvation can promote mitophagy and MDV production**.** Starvation triggers the activation of the energy sensor AMP-activated protein kinase (AMPK), which can activate the mammalian target of rapamycin (mTOR) or directly activate Autophagy Activating Kinase 1/2. This leads to the phosphorylation of Beclin1 and the promotion of mitochondrial autophagosome formation by Microtubule-associated protein 1 A/1B-light chain 3 (LC3). PTEN-induced putative kinase 1, located on the outer mitochondrial membrane, recruits Parkin from the cytoplasm, catalyzing the attachment of ubiquitin chains to outer mitochondrial membrane proteins. Under the action of LC3, autophagosomes are transported to lysosomes for degradation. During starvation, the production of MDVs also increases. The single-membrane MDVs labeled with mitochondria-associated protein ligase (MAPL) are directed to peroxisomes for degradation, while those labeled with translocase of outer mitochondrial membrane 20 (TOM20), as well as double-membrane MDVs labeled with pyruvate dehydrogenase (PDH), are transported to lysosomes for degradation. Notably, MDVs maintain membrane potential, contain ATP synthase subunits, and possess the ability to produce ATP

Mitophagy, a highly conserved self-regulatory mechanism, enables cells to selectively eliminate damaged or dysfunctional mitochondria. This evolutionarily conserved process plays a critical role in preserving mitochondrial quality control, maintaining bioenergetic homeostasis, and ensuring cellular survival through the precise removal of compromised organelles (Lu et al. 2023). While mitophagy plays a crucial role in cellular health, it can also reduce the overall number of mitochondria, potentially decreasing ATP production. Thus, it is essential to keep the balance between mitophagy and ATP production. Mitophagy occurs even under normal conditions but is significantly enhanced during periods of starvation (Mizushima and Klionsky 2007). Consequently, some researchers have enhanced mitophagy through starvation to explore the internal mechanism (Fig. 2).

This starvation-induced mitophagy is tightly regulated by energy-sensing pathways, with AMP-activated protein kinase (AMPK) emerging as a central molecular switch that integrates nutrient availability with mitochondrial quality control. AMPK can function as a bridge connecting starvation and mitophagy. The cellular energy sensor AMPK is activated when cellular energy levels are low (Li et al. 2023b). AMPK promotes autophagy by phosphorylating Unc-51 Like Autophagy Activating Kinase 1 (ULK1) and inhibiting mammalian target of rapamycin (mTOR) (Nacarkucuk et al. 2024). ULK1 and its isoform, Unc-51 Like Autophagy Activating Kinase 2 (ULK2), are highly expressed in adult cortical neurons and are involved in the regulation of mitophagy (Egan et al. 2011). The autophagic function is compromised by ULK1 loss (Titus et al. 2023). The Bcl-2 interacting protein (BECLIN1) complex, activated by ULK1 or ULK2, plays an upstream role in initiating autophagy. Subsequently, soluble N-ethylmaleimide-sensitive factor attachment protein receptors (SNARE) proteins facilitate the enclosure of defective mitochondria, leading to their fusion with lysosomes for degradation (Towers et al. 2021). The above outlines the general process of starvation-induced mitophagy and the key factors involved. In the following sections, the researchers provide an in-depth discussion of each of these factors (Yang et al. 2024b).

Given the central role of AMPK in coordinating mitophagy, pharmacological modulation of this energy-sensing kinase has emerged as a promising therapeutic strategy for age-related neuronal degeneration. Research has shown that AMPK activation promotes neuronal mitophagy and slows aging (Ulgherait et al. 2014). Metformin is a type 2 medication for diabetes that acts by activating AMPK, which changes downstream proteins and controls glucose and lipid metabolism (Yip et al. 2025). Additionally, research has demonstrated that, metformin can reduce brain ischemia–reperfusion injury in animal models by activating AMPK/ULK1/PTEN-induced putative kinase 1 (PINK1)/E3 ubiquitin-protein ligase parkin (Parkin) (Guo et al. 2023b). The PINK1/Parkin pathway represents a crucial regulatory mechanism for the activation of mitophagy, the selective autophagy of mitochondria (Lazarou et al. 2015). This is a landmark study on mitophagy. Parkin is an E3 ubiquitin ligase, and its terminus contains ubiquitin-like domains as well as RING-like domains (Popov 2022). However, excessive AMPK activation can have detrimental effects. Aβ42 oligomers, which are associated with early AD, have been found to hyperactivate AMPK, leading to synaptic loss via Mitochondrial Fission Factor (MFF) -dependent mitochondrial fission and ULK2-dependent mitophagy (Lee et al. 2022). MFF, located on the outer mitochondrial membrane, recruits dynamin-related protein 1 (DRP1), a key protein in mitochondrial fission (Sun et al. 2024a). This highlights the importance of mild modifications to energy metabolism to regulate AMPK activation. Over-activation of AMPK may result in mitochondrial fragmentation and accelerated mitochondrial dysfunction, as the process might fail to remove damaged mitochondria and accumulate aberrant protein effectively. Remarkably, new research indicates that AMPK may also limit ULK1 activity and autophagy (Park et al. 2023), suggesting that a complex role of AMPK beyond merely promoting autophagy. This dual role of AMPK in autophagy, depending on the context and stimuli, warrants further investigation to better understand its impact on neuronal health and disease.

### MDVs in Energy Metabolism

MDVs are small vesicles that originate from mitochondria, with diameters ranging from 70 to 150 nm. They are divided into two types: single-membrane and double-membrane MDVs (Mishra and Deep 2024). Single-membrane MDVs bud from the outer membrane of mitochondria and contain proteins from the intermembrane space, using TOM20 as a marker. Double-membrane MDVs originate from the inner membrane of the mitochondria containing proteins from the mitochondrial matrix, and use PDH as a marker (Soubannier et al. 2012). MDVs selectively transport damaged mitochondrial components, including oxidized protein aggregates, to lysosomes or peroxisomes for targeted degradation, thereby compartmentalizing the elimination of dysfunctional organellar elements while preserving overall mitochondrial integrity (König and McBride 2024). Unlike the processes of mitochondrial fission and fusion, the formation of MDVs is dependent on the PINK1/Parkin pathway, rather than DRP1 (Sugiura et al. 2014). This pathway is distinct from PINK1/Parkin-mediated mitophagy because MDV formation is independent of the autophagy-related protein Microtubule-associated protein 1 A/1B-light chain 3 (LC3) (Ryan et al. 2020). MDVs are constitutively produced under mitochondrial homeostasis and their formation increases during oxidative stress. It is worth mentioning that MDVs are rapidly generated a few minutes after being induced by oxidative stress, earlier than mitophagy, and are referred to as the first line of defense against oxidative stress (McLelland et al. 2014). This also proves the importance of MDVs in responding to oxidative stress. Given the rapid and dynamic nature of MDV biogenesis, researchers have developed innovative experimental approaches to dissect the molecular composition and functional roles of these vesicles under various metabolic conditions.

Currently, some researchers have conducted in-depth studies on MDVs by stimulating their production with antimycin A. In a study by Vasam et al., H9 C2 cardiomyocytes exposed to antimycin A underwent proteomic analysis, revealing a notable increase in proteins involved in the metabolism of FAs, amino acids, and TCA cycles within MDV cargo (Vasam et al. 2021). In addition to antimycin A, changes in energy metabolism substrates can also promote MDVs production. For instance, culturing cells in galactose, which restricts ATP synthesis predominantly through OXPHOS, has been shown to enhance MDV formation (Opstad et al. 2022). Although current studies have revealed changes in MDVs under different energy metabolic states, these investigations have yet to explore the mechanisms underlying MDV production during galactose adaptation. To address this gap, analyzing key drivers of MDVs could elucidate how their biogenesis is upregulated in response to altered energy metabolic conditions, which merits further investigation. The effect of starvation on MDV formation remains unclear. Although starvation induces mitophagy, which shares the PINK1/Parkin pathway with MDVs, it is still unknown which pathway is predominant at different stages of starvation, or whether there is a connection among their internal mechanisms. Recent research has proposed an additional function for MDVs beyond the transportation of aberrant proteins for degradation. Ben-Menachem et al. discovered that isolated MDVs from yeast have functional F1 Fo-ATP synthase complexes and membrane potential, enabling them to produce ATP independently (Hazan Ben-Menachem et al. 2023). This suggests that MDVs may transfer ATP-generating machinery from damaged mitochondria to healthy ones, thereby compensating for the reduced ATP production in dysfunctional mitochondria.

### Mitochondrial Quality Control in Cerebral Aging and Neurodegenerative Disorders

While the dynamic equilibrium between mitochondrial energy production and quality control sustains cellular homeostasis in healthy states, aging disrupts this balance through progressive MQC failure. Neurons, with their unparalleled bioenergetic demands and limited regenerative capacity, are particularly vulnerable to such age-related mitochondrial dysfunction. This section delineates the dysregulation of MQC pathways in age-related neurological deterioration and neurodegenerative disorders, providing a mechanistic framework to decipher the pathogenic cascades driving mitochondrial dysfunction.

Mitophagy, a critical pathway for the selective elimination of damaged mitochondria, represents a key regulatory process in neuronal homeostasis (Nikoletopoulou 2024). A central question arising in this context is how mitophagic activity is modulated within the naturally aging brain. A study has revealed that the level of mitophagy increases with aging in microglia and cerebellar granule cells. In the hippocampus, which regulates memory, mitophagy shows an increasing trend until middle age, followed by a decline (Rappe et al. 2024). This research indicates that the intensity of mitophagy does not uniformly change across different brain regions or cell types as aging progresses. In light of these changes, identifying region-specific regulatory mechanisms of mitophagy is crucial for intervening in the aging process. Notably, this study revealed that macroautophagy also exhibits age-related changes, further highlighting the importance of investigating autophagic mechanisms in aging. Although this study extensively analyzed the changes in mitophagy and macroautophagy across different ages and brain regions, it remains unclear whether these two processes interact synergistically to regulate specific brain areas or operate through distinct mechanisms. This question merits in-depth investigation. Deficiencies in mitophagy have been observed in hippocampal neuron samples from patients with AD (Fang et al. 2019). Restoring neuronal mitophagy has been shown to ameliorate cognitive decline in AD model Caenorhabditis elegans. Both Aβ and tau have been demonstrated to disrupt the normal process of mitophagy (Cummins et al. 2019). Research indicates that mitochondria are dynamic organelles, and the maintenance of a dynamic balance between mitochondrial fission and fusion is one of the key pathways for MQC. In aging astrocytes, dysregulation of mitochondrial fusion and fission pathways leads to mitochondrial fragmentation (Araujo et al. 2024). Mitochondrial fragmentation detrimentally attenuates ATP biosynthesis and perturbs mitochondrial homeostasis, ultimately culminating in mitochondrial dysfunction and consequent cellular pathophysiology (Chaplygina and Zhdanova 2025). Indeed, studies have also found that aging astrocytes can compensatorily upregulate mitochondrial biogenesis (Diniz et al. 2024), which may serve as a compensatory mechanism against mitochondrial fragmentation. The accumulation of mAPP and Aβ has been shown to increase mitochondrial fission and reduce fusion in hippocampal neurons, characterized by elevated levels of fission proteins Drp1 and Fis1, alongside decreased expression of fusion proteins Mitofusin 1, Mitofusin 2, and Optic Atrophy 1 (Reddy et al. 2018). Collectively, neurodegenerative disorders and aging are associated with mitochondrial dysfunction, wherein two critical MQC pathways—mitochondrial fission/fusion dynamics and mitophagy—are significantly compromised (Nibrad et al. 2025). Regrettably, no studies have been identified linking MDVs to aging or neurodegenerative pathologies, highlighting a critical gap in current research. This underscores the necessity for future investigations to explore MDV-mediated mechanisms, potentially uncovering novel therapeutic targets to modulate brain aging or mitigate neurodegenerative disease progression.

## Dietary Intervention and Brain Aging

Given the centrality of mitochondrial bioenergetic competence in combating neurodegeneration, nutritional modulation of MQC pathways emerges as a strategic therapeutic paradigm. Researchers have explored various dietary interventions, such as calorie restriction (CR), the ketogenic diet (KD), intermittent fasting (IF), the Mediterranean diet (MedDiet), targeted nutrient restriction, and antioxidant supplementation, to address age-related metabolic inflexibility in neurons.

By mechanistically bridging nutrient-sensing pathways with organelle-level quality control, these dietary regimens demonstrate unparalleled potential to recalibrate the energy-MQC nexus disrupted in brain aging. Current efforts focus on developing intervention of neuroprotective efficacy. These therapeutic interventions exhibit distinct molecular mechanisms and exert differential impacts on various hallmarks of aging. Furthermore, we will critically evaluate innovative strategies to optimize existing dietary intervention protocols, with particular emphasis on precision nutrition approaches and their potential for enhancing intervention efficacy.

### Caloric Restriction

CR involves reducing daily caloric intake by 20–30% while ensuring adequate nutrition. This intervention has shown promise in improving health and delaying aging, not only through weight management but also via other mechanisms. For instance, studies on rhesus monkeys, which are genetically similar to humans and age in a comparable manner, indicate that CR can serve as a form of treatment for age-related conditions (Mattison et al. 2017). A two-year CR intervention has successfully reduced cellular aging biomarkers in middle-aged, healthy individuals (Aversa et al. 2024). However, while CR has extended the lifespan of certain primates like gray mouse lemurs, it has also been linked to accelerated loss of gray matter in the brain, suggesting possible detrimental effects on brain integrity (Pifferi et al. 2018). However, the results of this study indicate that gray matter atrophy is not associated with changes in cognitive ability. Therefore, the specific negative implications of this alteration remain open for in-depth discussion. Additionally, since all animals in the study were male, and given the potential sex-based differences, further research on gray matter atrophy in female animals under CR is also warranted. These paradoxical effects of CR—its capacity to extend lifespan while posing neurological risks—have driven efforts to dissect its molecular underpinnings and identify context-specific therapeutic windows.

To investigate the underlying mechanisms of CR intervention, researchers have investigated the changes in various factors under CR. CR impacts mitochondrial function and energy metabolism by upregulating superoxide dismutase 2 and activating the AMPK/silent information regulator sirtuin 1/Peroxisome proliferator-activated receptor-gamma coactivator-1 alpha signaling pathway (Vo et al. 2024). Furthermore, CR may mitigate mitochondrial dysfunction and oxidative stress in the aging brain by enhancing the plasma membrane redox system (Hyun et al. 2006). Brains of individuals undergoing CR show increased activity of complexes I + III and complex IV, which helps enhance the mitochondrial respiratory rates (Amigo et al. 2017). These effects are beneficial for energy metabolism, as CR improves glucose uptake, glycolytic efficiency, local ATP supply, and reduces oxidative stress (Guo et al. 2023c). CR has been shown to enhance insulin sensitivity, improve glucose and mitochondrial homeostasis, delay the aging of beta cells, and restore the beta cell function in diabetic mice (Dos Santos et al. 2024). Insulin resistance in the central nervous system is closely related to age-related neurodegenerative changes (Yang et al. 2021), suggesting that CR can help protect neurons by reducing insulin resistance. Another benefit of CR is mitophagy (Bagherniya et al. 2018). Research by Ferreira-Marques et al., suggests that CR, along with caloric restriction-mimicking neuropeptides, promote mitophagy in rat cortical neurons via specific signaling pathways, such as the PI3 K/AKT/mTOR and ERK 1/2-MAPK pathways (Ferreira-Marques et al. 2021). Besides, CR can also reduce oxidative stress through upregulating the production of sirtuin proteins that regulate cellular metabolism and the cell cycle (Ziętara et al. 2022).In terms of neuronal health, CR has been found to delay brain aging by reducing inflammation in the hippocampus and promoting neurogenesis and neurotransmission involving monoamines and glutamate (Gillespie et al. 2016; Rojic-Becker et al. 2019). Experimental evidence indicates that CR exerts potent anti-inflammatory effects on the aging brain, with particular efficacy in restoring compromised neurogenic capacity. This therapeutic benefit is achieved through CR-mediated modulation of the inflammatory milieu in the subventricular zone (SVZ) niche of aged animal models (Dugan et al. 2023; Apple et al. 2019). Neuroinflammation can trigger chronic systemic inflammation through a series of pathogenic cascades, including circadian clock dysfunction, gut dysbiosis, and immunosenescence, which will lead to systemic inflammatory senescence (Jurcau et al. 2024). Research has demonstrated that CR exerts dual anti-inflammatory effects, mitigating both neuroinflammation and systemic inflammation. For instance, a recent study revealed that CR effectively attenuates acute systemic inflammation triggered by lipopolysaccharide (LPS), with particularly significant reductions in inflammatory markers observed in the hippocampus (da Silva et al. 2024). Interestingly, gender-specific responses to CR have been identified, with female subjects exhibiting greater longevity benefits compared to males under CR conditions (Mitchell et al. 2016). Further supporting this observation, studies in aged female mice have uncovered CR-induced modifications in hypothalamic neuronal activity and gene expression patterns, which may represent potential biomarkers of aging (Hajdarovic et al. 2022). While CR has demonstrated positive effects in delaying aging, its broader impacts on the body warrant careful evaluation. A recent study revealed that mice subjected to 40% CR exhibited several indicators of improved health, including reduced body temperatures, heightened hunger responses, and alterations in their immune repertoire (Di Francesco et al. 2024). These findings highlight the importance of weighing the potential benefits of CR against the risk of adverse effects, emphasizing the need for researchers to consider whether excessive CR might harm the body when designing CR-based interventions. Moreover, the implementation of CR in long-term dietary regimens presents challenges, particularly regarding patient compliance and the design of clinical studies. Short-term intervention studies have often been the focus, as long-term adherence to CR is difficult to maintain (DeBlauw et al. 2023; Clayton et al. 2016). Although the underlying processes are yet unknown, Azadian et al. observed significant neuroprotective effects in rats that were treated with 14 h of CR before cardiac arrest and resuscitation (Azadian et al. 2020), However, this study still has limitations. We noted that the CR protocol in this research was initiated at 6:00 PM. Given the differences in diurnal metabolic rhythms between humans and mice, whether such a short-term CR regimen can yield similar benefits in humans warrants further investigation. While the mechanisms behind this ultra-short CR intervention are not fully understood, it is a relatively simple approach that warrants further investigation.

### Ketogenic-Diet

The KD is a low-carbohydrate, high-fat diet designed to shift the body’s metabolism from using glucose as its primary source of energy to using fats (Marinescu et al. 2024). When carbohydrate intake is significantly reduced, the body enters a metabolic state known as ketosis. In ketosis, the liver converts fats into molecules called KBs, which can be used as an alternative energy source, especially by the brain. When glucose intake is limited, ketones can act as direct energy substrates for the brain through the TCA cycle (Elamin et al. 2017). Compared with the mice in the normal group, a higher content of fumarate can be detected in the hippocampus of middle-aged mice with KD intervention (Roslund et al. 2024). Fumarate is one of the products of the TCA cycle and can promote the expression of Nrf2 (Izuta et al. 2018). The activation of Nrf2 has been proven to enhance the antioxidant capacity of neurons (Sadovnikova et al. 2021). Therefore, this metabolic difference under KD intervention may also be one of the reasons for its beneficial effects on neurons. A recent study found that β-OHB, the main product of KD, can regulate the TCA cycle by modifying Lysine β-hydroxybutyrylation of TCA cycle-associated enzymes (Han et al. 2025). Moreover, this regulation mainly occurs in neurons rather than glial cells. This finding indicates that KD can promote the TCA cycle and ATP production (Han et al. 2024).

Beyond its metabolic effects, KD has emerged as a promising therapeutic strategy for neurodegenerative diseases, leveraging its dual capacity to enhance mitochondrial bioenergetics and suppress neuroinflammation. Originally developed to treat epilepsy (Ijff et al. 2016), KD is currently being investigated for the treatment of neurodegenerative diseases (Gough et al. 2021). The enhanced mitochondrial function and the reduced neuroinflammation are the underlying mechanisms. Research by Jiang et al., indicates that KD reduces inflammation in AD models by stifling the NF-κB signaling pathway, thereby alleviating symptoms (Jiang et al. 2023). In addition, investigations show that in 1-Methyl-4-phenyl-1,2,3,6-tetrahydropyridine (MPTP)-induced PD rats, a ketogenic diet high in medium-chain triglycerides (MCT) has demonstrated neuroprotective effects by restoring dopamine cell function and improving motor deficits through activating the Phosphoinositide 3-kinase/Phosphoinositide 3-kinase pathway, which reduces oxidative stress (Zhang et al. 2023a). MCT has also been shown to improve spatial memory in rats (Shcherbakova et al. 2023). Given that brain aging is associated with certain aspects of neurodegenerative disorders, KD has been demonstrated to lower mortality in middle-aged mice and increase the healthy lifespan of older mice (Newman et al. 2017). This underscores the significance of investigating the mechanisms underlying the KD to develop innovative strategies for ameliorating age-related decline. To elucidate these intrinsic mechanisms, a comprehensive analysis of KD-induced metabolic alterations is essential.

The production of KBs, particularly βOHB, is closely related to the effects of the ketogenic diet. Research has shown that βOHB supplementation lowers GABA and Glu levels in the prefrontal cortex of healthy adults, with a more pronounced effect in older adults (Hone-Blanchet et al. 2023), suggesting increased sensitive to KBs with age. Furthermore, βOHB promotes an anti-inflammatory response in microglial cells by increasing the expression of cytokine Interleukin-10 and decreasing levels of the pro-inflammatory cytokine Interleukin-17, leading to a shift toward the M2 anti-inflammatory phenotype (Polito et al. 2023). Along with that, βOHB enhances ATP supply and reduces ROS levels in glucose-deprived Neuro-2a cells, suggesting its potential as a neuroprotective agent (Chiang et al. 2023). It's interesting to note that adding nutrients to KBs doesn't always provide full benefits. For example, unrestricted ketogenic diets have been shown to increase mortality in mice, and supplementing the βOHB precursor 1,3-butanediol has been shown to increase middle-aged mortality in normal mice but extend lifespan in aged mice (Tomita et al. 2023). This calls into question the efficacy of the ketogenic diet. This suggest that KD can produce health-promoting KBs, its efficacy may depend on the precise ratios and timing of nutrient intake. Excessive dietary fat can exacerbate inflammation, oxidative stress, autophagy dysregulation, and accelerating the aging process (Hou et al. 2022). The modified Mediterranean ketogenic diet (MMKD), which focus on high-quality fats and a balanced carbohydrate intake while maintaining the features of the standard KD, has also gained popularity in recent years. Studies suggest that MMKD may help regulate GABA levels to ameliorate moderate cognitive impairment and modify gut metabolism, influencing biomarkers in cerebrospinal fluid (Nagpal et al. 2019; Dilmore et al. 2023).

### Intermittent Fasting

IF is a dietary intervention characterized by alternating periods of fasting and eating. Specifically, IF can be categorized into alternate-day fasting (ADF), time-restricted feeding (TRF), and periodic fasting (Elias et al. 2023). ADF involves 24 h of fasting followed by 24 h of unrestricted eating. TRF is usually structured with a daily fasting period, such as fasting for 16 h and eating within an 8-h window. Periodic fasting often involves fasting for specific days within a week, with a common protocol being 2 days of fasting and 5 days of eating within a week. IF has been shown to extend lifespan and improve health outcomes (Longo and Panda 2016). For example, in male rats, it has been observed to ameliorate age-related deficits in memory and motor performance (Singh et al. 2012). IF has demonstrated superior efficacy than CR in enhancing cognitive function and promoting neurogenesis (Dias et al. 2021). While these phenotypic benefits of IF are well-documented, emerging research is now elucidating the molecular circuitry through which fasting exerts its neuroprotective effects—spanning from metabolic reprogramming to inflammasome regulation—providing mechanistic insights that could optimize therapeutic applications.

The positive effects of IF on neurons occur through multiple ways. Research has demonstrated that in a mouse model of PD, IF has dopaminergic neuroprotective properties (Ojha et al. 2023), indicating that it could potentially be used as a treatment or preventive strategy for neurodegenerative diseases. IF also inhibits oxidative stress markers like malondialdehyde in chronic cerebral hypoperfusion models while enhancing antioxidant enzymes such as SOD (Rajeev et al. 2022). While preliminary findings suggest promising anti-aging effects of IF, substantial mechanistic investigations are still required to elucidate its temporal and tissue-specific impacts throughout the aging trajectory. Notably, ADF has demonstrated significant immunomodulatory properties through the suppression of NLRP3 inflammasome activation, resulting in attenuated secretion of downstream pro-inflammatory cytokines, particularly interleukin-1β (IL-1β) and interleukin-18 (IL-18) (Wu et al. 2023). Concurrently, ADF appears to enhance hippocampal neuroplasticity by promoting neurogenic processes, suggesting a dual mechanism of action involving both anti-inflammatory pathways and neural regeneration. This supports the idea that ADF is a non-pharmacological method that can mitigate neuroinflammation (Wu et al. 2023). However, some studies have discovered that no significant effect of IF on the growth of adult neural stem cells (NSCs) or on neurogenesis in mice, highlight the need to consider factors such as mouse gender, fasting protocol, and overall health in future research (Gabarró-Solanas et al. 2023).

While divergent findings exist regarding specific outcomes like neurogenesis, emerging evidence systematically highlights IF’s anti-aging potential through multi-layered molecular mechanisms. Notably, its modulatory effects on cellular autophagy and mitochondrial function are providing more integrated perspectives to explain its biological impacts. Regarding the internal mechanism, IF has also been shown to upregulate autophagy markers (Ntsapi and Loos 2021), decrease ROS production and reduce oxidative stress related cellular damage (Ntsapi and Loos 2021). ADF and TRF can upregulate autophagy factors like LC3 and Beclin and enhance the activity of ETC complexes I and III (Bhoumik et al. 2022), which supports the role of IF in mitigating brain aging and neurodegenerative diseases through improved mitochondrial function (Zhao et al. 2022). These findings pertain to the internal mechanisms underlying neuroprotection and reduction of oxidative stress. Apart from the previously known variations of IF, current research appears to have found that fruit flies can live a greater duration when subjected to intermittent time-restricted feeding (iTRF). This phenomenon occurs without any connection to the mechanisms linked to dietary restriction. To slow down the aging process, iTRF modifies autophagy linked to circadian rhythms (Ulgherait et al. 2021). This chrono-autophagic mechanism, while promising, warrants further mechanistic investigation to elucidate its neuroprotective potential, particularly regarding the maintenance of proteostasis and MQC in aging neurons.

After conducting in-depth research on the mechanism of IF, some researchers, considering practical applications, have compared the difficulty of implementing IF with that of CR. For example, considering from the perspective of patient compliance, IF is easier to implement than CR (Yap et al. 2024). After cerebral ischemia, IF pretreatment has been demonstrated to improve the nervous system, potentially in the form of growth differentiation factor 11 (GDF11)/activin-like kinase 5 (ALK5) signaling (Liu et al. 2023). Further research into GDF11's potential as an effective way to delay neurodegeneration should be conducted as it has an innate capacity to catalyze neurogenesis (Katsimpardi et al. 2014). Moving forward, understanding the varying mechanisms of different IF strategies and identifying appropriate applications for specific populations will be crucial areas of study.

### Specific Nutrient Restriction

Unlike CR, specific nutrient restriction (SNR) focuses on changing a single dietary component—such as glucose or amino acid restriction—rather than reducing total caloric intake. Many dietary restriction strategies operate through complex internal mechanisms, making it challenging to isolate the role of a particular nutrient. This approach to dietary restriction facilitates further research into the contributions of specific nutrients to health, offering significant assistance in formulating overall dietary restriction strategies. The AMPK isoform AP2 Associated Kinase-2a (AAK-2a) mediates the lifespan-extending effect of a glucose restriction (GR) diet in Caenorhabditis elegans, according to research conducted by Jin-Hyuck Jeong et al. The underlying mechanisms involve the enhancement of peripheral tissue membrane fluidity and the control of neuropeptide signaling by AAK-2a (Jeong et al. 2023). This study suggests that AMPK has effects beyond autophagy, as it did not link the lifespan extension caused by GR with autophagy as previously indicated. In addition to glucose restriction, it has recently been discovered that amino acid restriction is good for health. Branched—chain amino acids (BCAAs) mainly include leucine, valine, and isoleucine. Recently, a study has found that BCAAs restriction can extend the lifespan of Drosophila through histone modification in neurons (Weaver et al. 2023). Mechanistically, BCAAs restriction can influence metabolism by modulating the mechanistic target of rapamycin complex 1 (MTORC1) pathway to postpone senescence (Mansoori et al. 2025). MTORC1 is regarded as one of the driving factors of aging and can inhibit autophagy (Liu and Sabatini 2020). Interestingly, it has been found that MTORC1 can enhance the expression of glycolytic enzymes by upregulating hypoxia-inducible factor 1 alpha (Düvel et al. 2010). These findings suggest a potential regulatory role of BCAAs in glycolytic pathways within the context of cellular energy metabolism. However, the mechanistic relationship between BCAA inhibition and its subsequent effects on glycolytic flux regulation remains to be elucidated. Furthermore, the potential downstream consequences of such metabolic alterations on ATP biosynthesis in aging organisms warrant systematic investigation through well-designed experimental studies. According to Wei Zhang et al., halving the number of amino acids in food increases antioxidant capacity and lengthens the life span of Drosophila melanogaster (Zhang et al. 2023b). Certain amino acids have been the focus of research, such as isoleucine restriction, which has been demonstrated to extend mouse life expectancy (Aman 2023). Furthermore, consumption of too many meals high in methionine can cause mitochondrial malfunction and oxidative stress (Di Minno et al. 2011), which raises the risk of age-related neurodegenerative disorders and hyperhomocysteinemia (Pang et al. 2019). Researchers discovered that methionine restriction reduces oxidative stress and lengthens lifespan to mitigate the hazards associated with high methionine diets (Lail et al. 2023). Despite the considerable translational barriers associated with implementing SNR in clinical practice, this approach holds substantial promise for elucidating the mechanistic contributions of individual nutritional components to the molecular pathways underlying biological aging.

### Mediterranean Diet

The Mediterranean diet is a dietary pattern based on the eating habits of countries bordering the Mediterranean Sea, characterized by a high intake of vegetables, fruits, grains, legumes, nuts, and healthy fats such as olive oil, with moderate consumption of red meat, low-fat dairy products, and red wine. Research indicates that the Mediterranean diet enhances antioxidant capacity (Pastori et al. 2016), an effect attributed to the abundant vitamins found in vegetables and fruits. Specifically, vitamins E and C exhibit antioxidant properties that protect neurons (Visioli et al. 2022). Furthermore, the Mediterranean diet demonstrates efficacy in reducing inflammatory markers, characteristics that collectively support its potential as a non-pharmacological intervention to enhance neuronal function (Register et al. 2024). A key component of this dietary regimen is olive oil, which is primarily composed of oleic acid—a monounsaturated fatty acid that exerts neuroprotective effects by inhibiting free radical generation, attenuating oxidative stress, and improving insulin sensitivity (Tortajada-Pérez et al. 2025). Whole grains, serving as the primary carbohydrate source in the Mediterranean diet, exhibit a lower glycemic response compared to refined grains, which may contribute to the prevention of insulin resistance (Picone et al. 2024). In terms of neurocognitive impact, studies demonstrate that adherence to the Mediterranean diet is associated with reduced stroke risk (Ungvari et al. 2025). Middle-aged animal models adhering to this dietary pattern further show enhanced learning and memory performance, suggesting neuroprotective benefits (Solch-Ottaiano et al. 2024). The polyphenol-rich Mediterranean diet demonstrates potential in mitigating age-related cerebral atrophy (Kaplan et al. 2022). In patients experiencing cognitive decline, implementation of this dietary pattern correlates with reduced rates of cognitive deterioration and medial temporal lobe atrophy (Kuhn et al. 2024). Mechanistically, transcriptomic profiling of Mediterranean diet-associated cerebral changes reveals upregulated cortical expression of TCIM—a modulator of the Wnt/β-catenin signaling pathway, the pathway plays a critical role in neurogenesis, neuronal survival, and the regulation of synaptic plasticity (Palomer et al. 2019). These findings collectively suggest that the Mediterranean diet may enhance cognitive function through modulation of TCIM expression (Li et al. 2024b). Patients with AD exhibit elevated plasma levels of pro-inflammatory markers and reduced expression of TGFβ (Mocali et al. 2004). TGF-β, known to suppress T-cell and B-cell activity, plays a critical role in modulating inflammatory responses. Following intervention with the Mediterranean diet, AD patients demonstrate increased expression of TGF-β2, suggesting a potential neuroprotective mechanism mediated by this dietary regimen (Hernando-Redondo et al. 2024).

Despite these promising mechanistic insights, current investigations into the Mediterranean diet’s efficacy remain predominantly centered on non-neurodegenerative populations, leaving critical gaps in understanding its applicability and challenges in AD-specific contexts. However, a recent study investigating the application of this dietary regimen in AD patients revealed lower adherence rates among this population (Dominguez et al. 2024). These findings provide novel insights into the timing of Mediterranean diet implementation. Given the adherence challenges, intervention during the pre-symptomatic phase may be optimal. Nevertheless, to evaluate therapeutic efficacy, future studies should employ animal models of neurodegenerative diseases to assess molecular-level biomarker changes following Mediterranean diet administration.

### Other Interventions

Beyond dietary interventions, alternative approaches such as pharmacological strategies demonstrate neuroprotective potential (Visioli et al. 2022). Current research has explored the incorporation of antioxidants into nutritional regimens to combat aging processes and enhance mitochondrial protection (Bagh et al. 2011). Notably, the therapeutic efficacy of antioxidants exhibits significant variation depending on their specific biochemical properties and mechanisms of action. A recent study demonstrated that prolonged administration of an antioxidant supplement formulation comprising N-acetylcysteine, α-lipoic acid, and α-tocopherol effectively ameliorates age-associated mitochondrial dysfunction (Bagh et al. 2011). Specifically, the investigation revealed that this antioxidant regimen preserves Complex IV activity, thereby maintaining optimal electron transport chain function and ATP biosynthesis. The mitochondria-targeted antioxidant Mitoquinone (MitoQ), which can traverse the inner mitochondrial membrane to directly reduce ROS, has recently garnered significant attention (Smith and Murphy 2010). Research has demonstrated that MitoQ treatment exerts positive effects on inflammation, antioxidant enzymes, and NADPH oxidase expression (Jeong et al. 2021). Notably, NOX play crucial roles in cellular signaling and immune responses (Kračun et al. 2025). NOX2 serve as a mediator of oxidative stress, research findings demonstrated that Aβ triggers oxidative stress via NOX2 activation, leading to the development of cerebral hypometabolism in murine models. In recent years, there has been a growing research interest in utilizing plant-derived antioxidants for the treatment of neurodegenerative diseases (Liang et al. 2021). Notably, studies have demonstrated the neuroprotective effects of myrtle berry byproduct extracts in 6-hydroxydopamine (6-OHDA)-induced PC12 cells (Dessì et al. 2025). 6-OHDA, a hydroxylated analogue of dopamine, is widely recognized for its ability to induce severe oxidative stress and dopaminergic neuronal damage both in vitro and in vivo, making it a commonly used agent for PD model induction (Masoudi et al. 2014). However, an intriguing aspect of antioxidant research reveals that not all oxidative compounds confer lifespan extension or health benefits. Notably, excessive supplementation of β-carotene and vitamin E has been shown to adversely affect mammalian health (Visioli et al. 2022). Consequently, in the therapeutic application of antioxidants for neurodegenerative diseases, researchers should not focus solely on their antioxidative properties but also consider their impact on neuronal function and overall lifespan. Future investigations should emphasize not only the development of novel antioxidant compounds but also the optimization of dosage regimens. This comprehensive approach will facilitate the formulation of effective therapeutic strategies for neurodegenerative disorders (Table 2).

**Table 2:** Dietary interventions have distinct mechanisms and effects

Intervention	Category	Mechanisms	Effect
Caloric restriction	Low-caloric diet	Dampening inflammation in the HPC	Attenuating the age-related decline in SOR memory (Portero-Tresserra et al. 2023)
	Reducing daily calorie intake to ~ 25% of usual intake	Reducing inflammatory infiltrates and vasogenic edema	Prevent brain volume loss in signature regions in AD and MS (Rahmani et al. 2023)
	Low-caloric diet	Reinforcing the stem cell pool and downregulating inflammation	Slowing down of the detrimental effects of aging (Erbaba et al. 2023)
	Caloric restriction mimetic (3 Bromopyruvate)	Increasing the antioxidant biomarkers and enhancing the activities of electron transport chain complexes I and IV, activating autophagy	Protecting the neurons (Arya et al. 2022)
	Combination of calorie restriction with exercise	Decreasing metabolic disturbance and hippocampal oxidative stress	Diminishing the brain pathologies and cognitive decline (Pratchayasakul et al. 2022)
Ketogenic diet	< 0.5% carbohydrates, 10% protein, and 90% fat for 7 months	Activating synaptic signaling cascade, increasing synaptic support through biochemical pathways p-ERK, p-CREB, BDNF	Rescuing deficient Long-term potentiation (Di Lucente et al. 2024)
	84% fat, 11% protein and 5% Carbohydrates for 14 days	Protecting mtDNA in the ipsilateral part of the cortex	Reducing the negative effects of stroke (Gureev et al. 2023)
	93.5% fat, 4.7% protein, and 1.8% carbohydrate for 3 months	Activating Nrf2/HO-1 pathway, inhibiting neuroinflammation	Improving learning and memory in of APP/PS1 transgenic mice (Jiang et al. 2023a)
	90% ketogenic diets, 5% ketone ester, and 5% peanut butter	Increasing mitochondrial efficiency	Improving behavioral recognition memory (Saito et al. 2022)
	90.50% kcal fat and < 0.50% kcal carbohydrate for 8 weeks	Reducing dopaminergic neuron loss and neurons by microbiota–gut–brain axis	Reducing motor dysfunction in PD mouse model (Jiang et al. 2023b)
Intermittent fasting	Alternate-day fasting for 22 weeks	Downregulating LCN2, GAL3	Attenuating HFD-induced inflammation and memory deficits (Lee et al. 2024)
	Alternate-day Fasting for 2 weeks	Increasing BDNF and Glial cell-derived neurotrophic factor expression and the PI3 K and PKB	Protecting the Dopaminergic neurons in a MPTP mouse model of PD (Ojha et al. 2023)
	16 h of fasting every day for 4 months	Maintaining the integrity of the neurovascular structures, reducing microvascular Leakage and blood brain barrier dysfunction	Reducing vascular and neuronal pathologies following Vascular cognitive impairment (Rajeev et al. 2022)

HPC, hippocampus; SOR, spatial object recognition; AD, Alzheimer's disease; MS, multiple sclerosis; p-ERK, protein kinase R-like endoplasmic reticulum kinase; p-CREB, phosphorylated c-AMP response element-binding; BDNF, brain-derived neurotrophic factor; Nrf2, nuclear factor erythroid 2-related factor 2; HO-1, heme oxygenase 1; APP, amyloid precursor protein; PS1, presenilin 1; PD, Parkinson disease; HFD, high-fat diet; PI3 K, phosphatidylinositol-3-kinase and protein kinase B,PKB, protein kinase B; MPTP, 1-methyl-4-phenyl-1,2,3,6-tetrahydropyridine; LCN2, Lipocalin-2; GAL3, Galectin-3

## Conclusions and Future Perspectives

The pathogenesis of brain aging can be attributed to three interrelated pathological processes: the progressive accumulation of intracellular oxidative stress, dysregulation of energy metabolic networks, and the persistent deterioration of mitochondrial function. In contrast to previous reviews, this study positions MQC as a central hub, demonstrating into role in delaying brain aging and associated neurodegenerative diseases through the regulation of energy metabolic pathways, such as mitophagy and MDV trafficking. To address these pathological processes, therapeutic strategies should focus on three key targets: (1) restoring mitochondrial integrity by enhancing MQC; (2) reestablishing metabolic stress balance to stabilize cellular homeostasis; and (3) modulating critical nodes in metabolic networks to optimize bioenergetic efficiency. Notably, mechanistic insights into neuronal substrate metabolism provide a foundation for pathway-specific interventions. For instance, targeting MDV transport mechanisms may offer novel approaches to preserve mitochondrial quality, while quantitative mitophagic flux could reveal dynamic features of energy regulation.

While dietary restriction has shown promise in attenuating aging biomarkers and extending healthspan by modulating these pathways, its clinical application faces two critical challenges. First, individualized nutritional regimens based on metabolomic profiles must be developed, necessitating a deeper understanding of the interaction network between MQC and energy metabolism. Second, there is a need for the development of biomarker systems capable of real-time monitoring of mitochondrial functional integrity, allowing for dynamic adjustment in personalized interventions. Therefore, future research should focus on creating a comprehensive framework that integrates mechanistic elucidation, target validation, and intervention optimization. This framework will serve as the theoretical basis for developing therapeutic strategies aimed at combating brain aging through metabolic reprogramming.

## Data Availability

No datasets were generated or analysed during the current study.
